# Quantum Adversarial Transfer Learning

**DOI:** 10.3390/e25071090

**Published:** 2023-07-20

**Authors:** Longhan Wang, Yifan Sun, Xiangdong Zhang

**Affiliations:** Key Laboratory of Advanced Optoelectronic Quantum Architecture and Measurements of Ministry of Education, Beijing Key Laboratory of Nanophotonics & Ultrafine Optoelectronic Systems, School of Physics, Beijing Institute of Technology, Beijing 100081, China

**Keywords:** quantum transfer learning, quantum generative adversarial network, quantum machine learning, quantum computation

## Abstract

Adversarial transfer learning is a machine learning method that employs an adversarial training process to learn the datasets of different domains. Recently, this method has attracted attention because it can efficiently decouple the requirements of tasks from insufficient target data. In this study, we introduce the notion of quantum adversarial transfer learning, where data are completely encoded by quantum states. A measurement-based judgment of the data label and a quantum subroutine to compute the gradients are discussed in detail. We also prove that our proposal has an exponential advantage over its classical counterparts in terms of computing resources such as the gate number of the circuits and the size of the storage required for the generated data. Finally, numerical experiments demonstrate that our model can be successfully trained, achieving high accuracy on certain datasets.

## 1. Introduction

Machine learning (ML) methods have successfully been applied to various fields such as speech recognition, visual object recognition, and object detection [1,2]. In recent years, ML research has been extended to applications in more complicated but ordinary scenarios, such as situations involving datasets belonging to different domains. The predicament in these scenarios is that the ML model trained on a dataset in one domain does not often work for tasks in a different domain of interest. One resource-consuming strategy used to solve the above issue is transfer learning (TL) [3,4,5,6,7,8], which estimates the usefulness of the knowledge learned in a source domain and transfers it to help the learning task in a target domain. However, conventional TL usually lacks efficiency when the distribution of the target domain data is completely different from the source domain. Recently, an adversarial transfer learning (ATL) method has been proposed to solve the issue. Such an ML scheme has the potential for a broad range of applications; it has been proven to be beneficial in areas such as natural language processing, autonomous cars, robotics, and image understanding [9,10,11,12,13,14,15,16,17,18,19].

**Figure 1:**
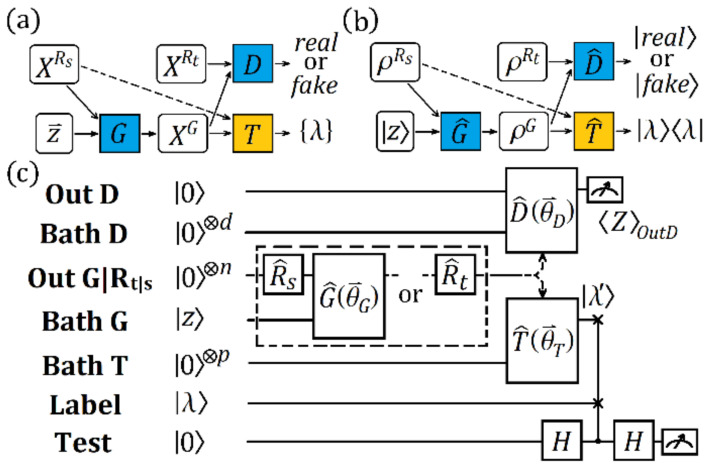
(**a**) Model architecture of ATL. The generator G produces a data sample $X^{G}$ on a source data sample $X^{R_{s}}$ and a noise vector $\vec{z}$. The discriminator D distinguishes $X^{G}$ from the target data sample $X^{R_{t}}$, assigning the judgement as “real” or “fake”. The classifier T assigns task-specific labels $\left\{\lambda \right\}$ to fake data sample *X^G^*. Note that source data sample $X^{R_{s}}\;$ is only accepted into the next step when the efficiency of the training is increased, as marked by the dashed arrow. (**b**) The corresponding QATL scheme. The data samples (${X^{R_{s}}}_{s}$, $X^{R_{t}}$, and $X^{G}$ ) are encoded by the quantum state $X^{R_{s}}$ ($\rho^{R_{s}}$, $\rho^{R_{t}}$, and $\rho^{G}$ ), respectively. The functioning of G, D, and T is implemented by the quantum operators $\hat{G}$, $\hat{D}$, and $\hat{T}$, respectively. The judgement of $\hat{D}$ is given by a quantum state ($|real\rangle \;$ or $|fake\rangle$ ) and the label is also encoded by $|\lambda \rangle$. (**c**) The quantum circuit of QATL. The qubit numbers of the registers **Bath D**, **Out G|R_t|s_**, and **Bath T** are *d*, *n*, and *p*, respectively. The registers **Out D** and **Test** both contain one qubit. Their qubits are initialized as $|0\rangle$. The register **Bath G** stores an m-qubit random state $|z\rangle$ generated by the environmental coupling. The register Label has the initial state $|\lambda \rangle$. ${\hat{R}}_{t}$, ${\hat{R}}_{s}$, $\hat{G}\left({\vec{\theta }}_{G}\right)$, $\hat{D}\left({\vec{\theta }}_{D}\right)$, and $\hat{T}\left({\vec{\theta }}_{T}\right)$ represent the unitary operators. The dashed box marks an option of applying two types of setups. H is the Hadamard gate. $\langle Z_{outD}\rangle$ is the expectation value of operator Z, as defined in the main text.

The basic idea of the ATL method is to introduce generative models to bridge the gap between the datasets of different domains. This method is a new version of ML and cannot be provided by simply combining methods such as generative adversarial networks (GANs) [20,21,22] and TL. As schematically shown in Figure 1a, ATL controls the samples of a given source domain dataset and a target domain dataset, denoted as $X^{R_{s}}$ and $X^{R_{t}}$, respectively. A generator G is employed to produce a fake sample $X^{G}$ using $X^{R_{s}}$ and a noise vector $\vec{z}$. $X^{G}$ and $X^{R_{t}}$ are then sent to a discriminator D to judge the probabilistic likelihood. The general purpose of training G and D is an adversarial game. Generator G is required to generate $X^{G}$ as close as possible to the target data sample $X^{R_{t}}$, cheating the discriminator D. Discriminator D attempts to accurately distinguish $X^{G}$ from $X^{R_{t}}$ and avoid being cheated. The game finally reaches the Nash equilibrium [23] after the parameters of the model are optimized. In cases of classification, a classifier T is correctly applied to label $X^{G}$ according to the label of $X^{R_{s}}$. In certain cases, the source domain dataset is also employed as the input of *T*, which increases the efficiency of the training. The key ingredient in such a scheme is the cost function of the model, which finally converges to an equilibrium point.

Despite the success of the above-mentioned method, the increasing requirements of data processing present a significant challenge to all computing strategies, including ML. As quantum computing provides an opportunity to overcome this challenge, researchers have considered addressing ML tasks using quantum machines [24,25,26,27,28,29,30,31,32,33,34,35,36,37,38,39,40,41,42,43,44,45,46,47,48,49,50,51,52,53,54,55,56,57]. Proposed quantum machine learning algorithms include quantum support vector machines [29,30], quantum deep learning [32,33,34,35,36,37,38], quantum Boltzmann machines [39,40], quantum generative adversarial learning [41,42,43,44,45], and quantum transfer learning (QTL) [47,48,49,50,51]. A quantum counterpart of ATL has not yet been provided; this could exhibit an advantageous performance in cross-domain learning tasks. To propose such a counterpart is complex because a suitable quantum cost function must be established so that the quantum adversarial game for knowledge transfer can finally converge to an equivalent point, as in classical cases. How to develop such a function in the quantum regime and how to obtain a quantum version of ATL remain unknown.

In this paper, we demonstrate how we solved the above problem, and we propose quantum adversarial transfer learning (QATL) schemes. The training process of our QATL was equivalent to an adversarial game of a quantum generator and a quantum discriminator, and we demonstrate that an equilibrium point also existed in the model. Specifically, a quantum cost function for adversarial training is provided; a measurement-based judgment of the data label and a quantum subroutine to compute the gradients are also discussed in detail. We prove that our proposal has an exponential advantage over classical counterparts in terms of computing resources such as the gate number of the circuits and the size of the storage for the generated data. This is of benefit to the transfer of complicated knowledge, during which a module is extensively called upon and a large amount of data are generated. We applied this scheme to a classification task based on the Iris dataset and the states on a Bloch sphere to prove that an extremely high classification accuracy could be achieved using this method.

## 2. Materials and Methods

Our QATL scheme is shown in Figure 1b. Here, the density matrix of the state that encodes a source (target) domain data sample is denoted by $\rho^{R_{s}}$ ($\rho^{R_{t}}$). A unitary operator is employed as the quantum generator $\hat{G}$, whose parameters are denoted by a vector ${\vec{\theta }}_{G}$. It operates on the state $\rho^{R_{s}}$ and a quantum noise state $|z\rangle \langle z|$, outputting a *fake* data sample $\rho^{G}$. Another unitary operator, parameterized by vector ${\vec{\theta }}_{D}$, is used as the quantum discriminator $\hat{D}$. It operates on the state $\rho^{G}$ generated by $\hat{G}$ and the state $\rho^{R_{t}}$; it then outputs the state $|real\rangle \langle real|$. If it operates on $\rho^{G}$, the state is $|fake\rangle \langle fake|$. As with ATL, the general training purpose of QATL is to maximize the probability of $\rho^{G}$ passing the test of discriminator $\hat{D}$, simultaneously minimizing the probability of $\hat{D}$ being cheated if $\rho^{G}$ is fake. The optimization of the parameters ${\vec{\theta }}_{G}$ and ${\vec{\theta }}_{D}$ are addressed when the above quantum adversarial game reaches the Nash equilibrium. We considered the classification task and applied a unitary operator parameterized by ${\vec{\theta }}_{T}$ as a quantum classifier $\hat{T}$ [58,59,60,61]. This provided a mapping of the data sample ($\rho^{G}$ or $\rho^{R_{s}}$) to a label state $L=|\lambda^{\prime }\rangle \langle \lambda^{\prime }|$; ${\vec{\theta }}_{T}$ was updated during the training.

Next, we discuss the state evolution of the above scheme on a circuit and demonstrate how an equilibrium point of the game was reached. The quantum circuit of Figure 1b is displayed in Figure 1c. The whole circuit ran on seven quantum registers. The register **Out G|R_t|s_** containing *n* qubits encoded the target data, source data, or the generated data. The encoding of the target (source) data was described by the operator ${\hat{R}}_{t}$ (${\hat{R}}_{s}$), which acted on the register **Out G|R_t|s_**. The register **Bath G**, containing *m* qubits, encoded the quantum noise state $|z\rangle \langle z|$. The generator $\hat{G}\left({\vec{\theta }}_{G}\right)$ operated on **Out G|R_t|s_** and **Bath G**, producing the generated data. The register **Bath D**, containing *d* qubits, was employed as the internal workspace of the discriminator $\hat{D}$, whose outputs ($|real\rangle \langle real|$ or $|fake\rangle \langle fake|$) were stored by the register **Out D**. The register **Bath T** contained *p* qubits and was employed as the workspace of the classifier $\hat{T}$. The label state $|\lambda^{\prime }\rangle$ outputted by $\hat{T}$ was compared with the single qubit label state $|\lambda \rangle$ stored by the register **Label**. The register **Test** was used to perform the estimation of the likelihood of $|\lambda^{\prime }\rangle$ and $|\lambda \rangle$, so that $\rho^{G}$ could be properly labeled. All qubits were initialized to be $|0\rangle$, except for those in **Bath G** and **Label**. The initial state of the quantum circuit shown in Figure 1c could then be denoted by

(1) $$\rho^{in}\left(z\right)=|0\rangle {\langle 0|}^{⊗\left(1+d+n\right)}⊗|z\rangle \langle z|⊗|0\rangle {\langle 0|}^{⊗p}⊗|\lambda \rangle \langle \lambda |⊗|0\rangle \langle 0|,$$

where ⊗ is the tensor product and $|0\rangle {\langle 0|}^{⊗p}$ is the tensor product of *p* $|0\rangle \langle 0|$s; this was also applicable for other similar terms. The unitary operators of such a state encoding the source domain data, target domain data, and generated data were denoted by $U_{R_{s}}$, $U_{R_{t}}$, and $U_{G}\left({\vec{\theta }}_{G}\right)$, respectively. They were given by

(2) $$U_{R_{s}}=I^{⊗\left(1+d\right)}⊗{\hat{R}}_{s}⊗I^{⊗\left(m+p+s+1\right)},$$

(3) $$U_{R_{t}}=I^{⊗\left(1+d\right)}⊗{\hat{R}}_{t}⊗I^{⊗\left(m+p+s+1\right)},$$

(4) $$U_{G}\left({\vec{\theta }}_{G}\right)=I^{⊗\left(1+d\right)}⊗\hat{G}\left({\vec{\theta }}_{G}\right)⊗I^{⊗\left(p+s+1\right)}$$

where I represents the 2×2 identity matrix. The states $\rho^{R_{s}}$ and $\rho^{R_{t}}$ were then given by

(5) $$\rho^{R_{s}}=U_{R_{s}}\rho^{in}\left(0\right)U_{R_{s}}^{†},$$

(6) $$\rho^{R_{t}}=U_{R_{t}}\rho^{in}\left(0\right)U_{R_{t}}^{†}.$$

We set **Bath G** to be ${|0\rangle }^{⊗m}$ instead of rotating it to a random quantum state with extra control. A random quantum state can naturally be generated by the entanglement of the register state and environmental degrees [44]. After $|z\rangle \langle z|$ was generated according to the requirements, the state $\rho^{G}$ could be expressed by

(7) $$\rho^{G}=U_{G}\left({\vec{\theta }}_{G}\right)\rho^{R_{s}}U_{G}^{†}\left({\vec{\theta }}_{G}\right).$$

The discriminator $\hat{D}\left({\vec{\theta }}_{D}\right)$ was applied to estimate the likelihood of $\rho^{G}$ and $\rho^{R_{t}}$. The unitary operator of $\hat{D}\left({\vec{\theta }}_{D}\right)$ was given by

(8) $$U_{D}\left({\vec{\theta }}_{D}\right)=\hat{D}\left({\vec{\theta }}_{D}\right)⊗I^{⊗\left(m+p+s+1\right)}.$$

Therefore, the resultant states of $\rho^{R_{t}}$ and $\rho^{G}$ after being operated by $U_{D}\left({\vec{\theta }}_{D}\right)$ were given by

(9) $$\rho^{DR_{t}}\left({\vec{\theta }}_{D}\right)=U_{D}\left({\vec{\theta }}_{D}\right)\cdot \rho^{R_{t}}\cdot U_{D}^{†}\left({\vec{\theta }}_{D}\right),$$

(10) $$\rho^{DG}\left({\vec{\theta }}_{D},{\vec{\theta }}_{G},z\right)=U_{D}\left({\vec{\theta }}_{D}\right)\cdot \rho^{G}\left({\vec{\theta }}_{G},z\right)\cdot U_{D}^{†}\left({\vec{\theta }}_{D}\right).$$

The expectation value of operator $Z\equiv |real\rangle \langle real|-|fake\rangle \langle fake|$ could be measured on the register **Out D**. Such a value was close to 1 when $\hat{D}\left({\vec{\theta }}_{D}\right)$ generated the state $|real\rangle \langle real|$; it was close to −1 when $\hat{D}\left({\vec{\theta }}_{D}\right)$ outputted the state $|fake\rangle \langle fake|$.

The quantum classifier $\hat{T}$ we considered was forced to output the label of $\rho^{G}$ according to its closeness to $\rho^{R_{s}}$. We also introduced this method to judge whether the label was correct. The unitary operator of $\hat{T}$ was defined by

(11) $$U_{T}\left({\vec{\theta }}_{T}\right)=I^{⊗\left(1+d\right)}⊗{\hat{T}}_{1}\left({\vec{\theta }}_{T_{1}}\right)⊗I^{⊗m}⊗{\hat{T}}_{2}\left({\vec{\theta }}_{T_{2}}\right)⊗I^{⊗\left(1+s\right)}$$

where ${\hat{T}}_{1}$ and ${\hat{T}}_{2}$ are two unitary operators on **Out G|R_t|s_** and **Bath T**, respectively. Their parameters were denoted by ${\vec{\theta }}_{T_{1}}$ and ${\vec{\theta }}_{T_{2}}$ correspondingly. Thus, ${\vec{\theta }}_{T}=\left({\vec{\theta }}_{T_{1}},{\vec{\theta }}_{T_{2}}\right)$. The resultant states of $\rho^{R_{s}}$ and $\rho^{G}$ after being operated by $U_{T}\left({\vec{\theta }}_{T}\right)$ were given by

(12) $$\rho^{TR_{s}}\left({\vec{\theta }}_{T},{\vec{\theta }}_{D},z\right)=U_{T}\left({\vec{\theta }}_{T}\right)\cdot \rho^{R_{s}}\left({\vec{\theta }}_{G},z\right)\cdot U_{T}^{†}\left({\vec{\theta }}_{T}\right),$$

(13) $$\rho^{TG}\left({\vec{\theta }}_{T},{\vec{\theta }}_{D},z\right)=U_{T}\left({\vec{\theta }}_{T}\right)\cdot \rho^{G}\left({\vec{\theta }}_{G},z\right)\cdot U_{T}^{†}\left({\vec{\theta }}_{T}\right).$$

One qubit of the above output state was the predicted label $|\lambda^{\prime }\rangle$ by $\hat{T}$. When comparing it with the label $|\lambda \rangle$ of the input $\rho^{R_{s}}$ by the swap test circuit [62] (which was composed of two Hadamard gates and one Fredkin gate, as shown in Figure 1c), the label of $\rho^{G}$ was set to be $|\lambda \rangle$ if $|\lambda^{\prime }\rangle$ and $|\lambda \rangle$ were close enough. The closeness of $|\lambda^{\prime }\rangle$ and $|\lambda \rangle$ could be estimated by the measurement of the register **Test**. The probability of the register **Test** being in state $|0\rangle$ was given by

(14) $$P_{0}=\frac{1}{2}\left(1+P_{c}\right)$$

where $P_{c}$ is the overlap of $|\lambda^{\prime }\rangle$ and $|\lambda \rangle$, defined by $P_{c}=Tr\left\{|\lambda \rangle \langle \lambda |L\left({\vec{\theta }}_{T},{\vec{\theta }}_{G},z\right)\right\}$. Therefore, $P_{c}$ could be estimated by $P_{c}=2P_{0}-1$. In specific cases, we could set a suitable boundary for accepting $|\lambda \rangle$ to be the label of $\rho^{G}$ (see the example below). Finally, the training of the quantum model could be performed by finding the following minimax point of the quantum cost function $V\left({\vec{\theta }}_{G},{\vec{\theta }}_{D},{\vec{\theta }}_{T}\right)$, given by

(15) $$\tilde{V}\left({\vec{\theta }}_{G},{\vec{\theta }}_{D},{\vec{\theta }}_{T}\right)=\frac{1}{M}\overset{M}{\underset{k=1}{\sum }}\left[\frac{1}{2}+\frac{1}{2}\cos^{2}\phi \mathrm{Tr}\left\{Z\rho_{k}^{DR_{t}}\left({\vec{\theta }}_{D}\right)\right\}\;\;\;\;\;-\frac{1}{2}\sin^{2}\phi \mathrm{Tr}\left\{Z\rho_{k}^{DG}\left({\vec{\theta }}_{D},{\vec{\theta }}_{G},z\right)\right\}\;\;\;\;\;\;\;\;\;\;\;\;-\mathrm{Tr}\left\{|\lambda_{k}\rangle \langle \lambda_{k}|\left[L_{k}\left({\vec{\theta }}_{T},{\vec{\theta }}_{G},z\right)+L_{k}^{R_{s}}\left({\vec{\theta }}_{T}\right)\right]\right\}\right].$$

The minimax point of $\tilde{V}\left({\vec{\theta }}_{G},{\vec{\theta }}_{D},{\vec{\theta }}_{T}\right)$ was obtained by tuning ${\vec{\theta }}_{G}$ and ${\vec{\theta }}_{T}$ to the minimum and tuning ${\vec{\theta }}_{D}$ to the maximum. The subscript k represents the different samples fed into the model. The number of the samples is denoted by M. $L_{k}^{R_{s}}\left({\vec{\theta }}_{T}\right)$ is the label state outputted by $\hat{T}$ when the input was the source domain data sample $\rho_{k}^{R_{s}}$. Such a term provides a bias; this could increase the training efficiency. The angle ϕ is the parameter used to adjust the weight of the target domain data and the generated data in the cost function, and it could be set according to the requirements of the specific tasks. A usual form was given by considering that they were equally weighted. By setting ϕ=π/4, we obtained

(16) $$V\left({\vec{\theta }}_{G},{\vec{\theta }}_{D},{\vec{\theta }}_{T}\right)=\frac{1}{M}\overset{M}{\underset{k=1}{\sum }}\left[\frac{1}{2}+\frac{1}{4}\mathrm{Tr}\left\{Z\rho_{k}^{DR_{t}}\left({\vec{\theta }}_{D}\right)\right\}-\frac{1}{4}\mathrm{Tr}\left\{Z\rho_{k}^{DG}\left({\vec{\theta }}_{D},{\vec{\theta }}_{G},z\right)\right\}\;\;\;-\mathrm{Tr}\left\{|\lambda_{k}\rangle \langle \lambda_{k}|\left[L_{k}\left({\vec{\theta }}_{T},{\vec{\theta }}_{G},z\right)+L_{k}^{R_{s}}\left({\vec{\theta }}_{T}\right)\right]\right\}\right].$$

The minimax optimization problem of Equation (16) was implemented by alternating between two steps. In the first step, we updated the discriminator and the classifier parameters ${\vec{\theta }}_{D}$ and ${\vec{\theta }}_{T}$ whilst keeping the generator parameter ${\vec{\theta }}_{G}$ fixed. In the second step, we fixed ${\vec{\theta }}_{D}$ and ${\vec{\theta }}_{T}$ and updated ${\vec{\theta }}_{G}$. We used the gradient descent method to update parameters ${\vec{\theta }}_{G}$, ${\vec{\theta }}_{D},$ and ${\vec{\theta }}_{T}$, respectively. The values of ${\vec{\theta }}_{G}$, ${\vec{\theta }}_{D},$ and ${\vec{\theta }}_{T}$ given by the lth update were denoted by $\vec{\theta }{}_{G}^{l}$, $\vec{\theta }{}_{D}^{l}$, and $\vec{\theta }{}_{T}^{l}$, respectively, with integer l=1,2,…; $\vec{\theta }{}_{G}^{0}$, $\vec{\theta }{}_{D}^{0}$, and $\vec{\theta }{}_{T}^{0}$ as the initial values. Hence, the update rule was expressed by

(17) $$\vec{\theta }{}_{G}^{l+1}=\vec{\theta }{}_{G}^{l}-\chi_{G}^{l}\nabla_{{\vec{\theta }}_{G}}V\left({\vec{\theta }}_{G},{\vec{\theta }}_{D},{\vec{\theta }}_{T}\right),\;\vec{\theta }{}_{D}^{l+1}=\vec{\theta }{}_{D}^{l}-\chi_{D}^{l}\nabla_{{\vec{\theta }}_{D}}V\left({\vec{\theta }}_{G},{\vec{\theta }}_{D},{\vec{\theta }}_{T}\right),\;\vec{\theta }{}_{T}^{l+1}=\vec{\theta }{}_{T}^{l}-\chi_{T}^{l}\nabla_{{\vec{\theta }}_{T}}V\left({\vec{\theta }}_{G},{\vec{\theta }}_{D},{\vec{\theta }}_{T}\right),$$

where $\chi_{G}^{l}$, $\chi_{D}^{l}$, and $\chi_{T}^{l}$ are the learning rates. In the above scheme, we used the variational quantum circuit in [44] and [58] to implement the generator $\hat{G}$, the discriminator $\hat{D},$ and the classifier $\hat{T}$, as shown in Figure 1c. In [44], each quantum gate of a quantum circuit corresponded with a parameter of the quantum circuit. The number of quantum gates was polynomial in the number of qubits; that is, 3/2*Lq*. *L* was the number of layers of quantum circuits and *q* was the number of qubits. Therefore, the number of parameters was also 3/2*Lq*. In [58], each quantum gate of the circuit had three parameters. The number of quantum gates was polynomial in the number of qubits, which was 2*Lq +* 1. Therefore, the number of parameters was 6*Lq +* 3. In our scheme, the total number of quantum gates was bound by those operating on the register **Out G|R_t|s_**, which was the sum of the number of quantum gates employed by the generator–discriminator sequence or the classifier. Based on the gate number of the above two circuits for the unitary operations we considered, the sum of the number of quantum gates remained polynomial in the number of qubits. We added two registers, **Label** and **Test**. Their gate number was constant and their impact on the complexity was negligible. In the process of obtaining the quantum cost function, the number of gates and the number of parameters of our scheme were polynomial in their number of qubits, which required exponentially fewer resources than the classical counterparts [10]. This property potentially benefits future applications for tasks involving complicated data structures. In general, if the information in a target dataset is insufficient, a large amount of data must be generated to effectively transfer the knowledge required to improve the training of the target dataset. This indicates a large storage requirement for the data and extensive calls of the modules. Otherwise, the gap between the features of different datasets is not bridged and the helpful information required to improve the training process is not captured and transferred. The improvement in computing resources by our QATL could loosen the requirements on storage and boost the efficiency of running the subroutines of the module, facilitating the execution of tasks.

To provide a more direct connection between the computation of the cost function and the updates of the parameters, we also applied two quantum circuits to calculate the gradients. Their theoretical scheme and circuit design are shown in Section SI of the Supplementary Materials. This scheme could be implemented on the recent quantum experimental platform because it was mainly based on variational quantum algorithms (VQAs) [63,64,65,66].

## 3. Results

To demonstrate the feasibility of QATLs, we considered their application in cases in which the probability distributions of the data in the two domains were different. The examples used the Iris dataset [67] and the states of the Bloch sphere.

The main task we considered was a classification of the Iris dataset in a numerical simulation. We chose two types of Iris flowers from the Iris dataset: *versicolor* and *virginica*. Each type contained 50 data samples. All data samples contained four attributes: sepal length (SL), sepal width (SW), petal length (PL), and petal width (PW). We considered encoding the four attributes based on the amplitudes of a two-qubit state (amplitude-encoding strategy [47]). The source domain dataset was composed of 40 data samples; 20 were picked from the *Iris versicolor* data samples and labeled $|0\rangle \langle 0|$. The other 20 were picked from *Iris virginica* data and labeled $|1\rangle \langle 1|$. The target domain dataset was also a 20/20 set chosen from samples other than those applied to the source domain dataset and without labeling. The last 10 data samples of both types were used to obtain a test dataset to check the performance of the model. The classification task labeled the target domain data samples using differently distributed source domain data. To enlarge the statistical difference of the source domain dataset and the target domain dataset and to improve the difficulty of classifying Iris flowers, we preprocessed the data samples by reducing the SL by 7 cm, the SW by 3 cm, the PL by 4 cm, and set the value of PW to 0. An illustration of the distribution of the data can be found in [67].

**Figure 2:**
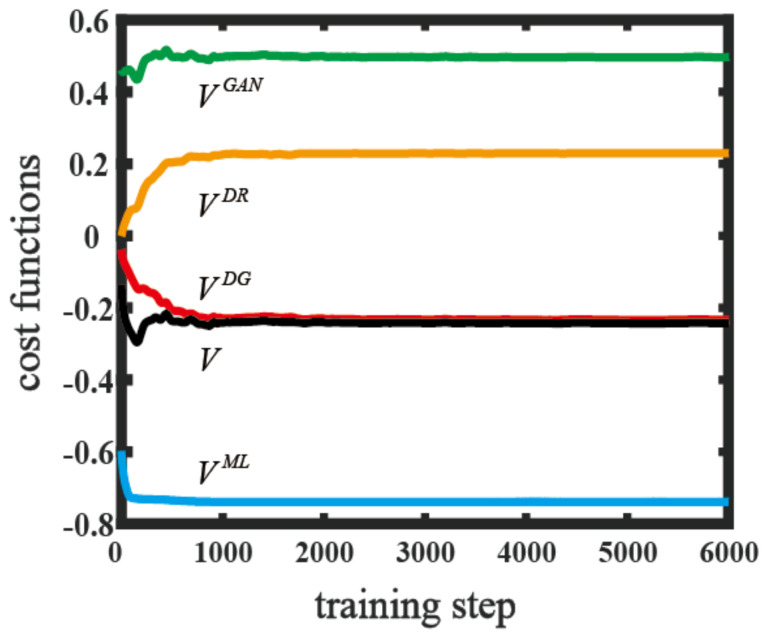
The values of quantum cost functions as a function of the training step on the Iris dataset. *V* is the total cost function. *V^ML^*, *V^GAN^*, *V^DG^*, and *V^DR^* represent various components of the cost function and are indicated by blue, green, red, and orange lines, respectively.

To show the convergence of terms of the cost function on the above datasets, we plotted the cost function V and its components as a function of the training step in Figure 2a. The cost function V converged to −0.217. The component $V^{DG}$ was defined as $V^{DG}=-1/4M\cdot \sum_{k=1}^{M}\mathrm{Tr}\left\{Z\rho_{k}^{DG}\left({\vec{\theta }}_{D},{\vec{\theta }}_{G},z\right)\right\}$, which represented the average probability of $\rho_{k}^{DG}\left({\vec{\theta }}_{D},{\vec{\theta }}_{G},z\right)$ being $|real\rangle \langle real|$, weighted by −1/4. $V^{DR}=1/4M\cdot \sum_{k=1}^{M}\mathrm{Tr}\left\{Z\rho_{k}^{DR_{t}}\left({\vec{\theta }}_{D}\right)\right\}$ represented the average probability of $\rho_{k}^{DR_{t}}\left({\vec{\theta }}_{D}\right)$ being $|real\rangle \langle real|$, weighted by −1/4. $V^{GAN}\equiv 1/2+V^{DG}+V^{DR}$; this reflected the capability of $\hat{D}$ to identify the generated data. $V^{DG}$, $V^{DR},$ and $V^{GAN}$ converged to 1/4, −1/4, and 1/2, respectively; thus, $\hat{D}$ could not provide a fair judgement and designate all the density matrices as $|real\rangle$. The average probability of matching the label with the generated data was given by $V^{ML}=-1/M\cdot \sum_{k=1}^{M}\mathrm{Tr}\left\{|\lambda_{k}\rangle \langle \lambda_{k}|\left[L_{k}\left({\vec{\theta }}_{T},{\vec{\theta }}_{G},z\right)+L_{k}^{R_{s}}\left({\vec{\theta }}_{T}\right)\right]\right\}$, which eventually reached −0.739. As Figure 2 clearly demonstrates, the cost function and its components converged to stable values in only 1500 training steps. We tested the trained model on the classification using the test dataset mentioned above. The classification accuracy reached 95%. The results demonstrated that an equilibrium point existed for the proposed cost function and the QATL model was workable on the dataset. Due to the generative model and adversarial training, the gap of knowledge transfer between the differently distributed datasets was effectively bridged; therefore, the accuracy of the classification was largely improved compared with previous quantum algorithms [51]. The method is shown in Appendix A. We also applied our scheme to the classification of the states of the Bloch sphere; detailed descriptions are shown in Section SII of the Supplementary Materials. A high-efficiency classification accuracy was also observed.

## 4. Conclusions

We demonstrated an efficient quantum machine learning scheme for cross-domain learning problems—QATL—which was a quantum counterpart of the recent and well-received ATL. Using the well-defined quantum cost function, an adversarial training process was applied to the transfer of knowledge, which was independent of specific tasks. Our numerical experiments demonstrated that the QATL model could successfully be trained and outperformed state-of-the-art algorithms in the same tasks. The complexity of the algorithm was logarithmic in terms of the number of quantum gates and training parameters, showing an advance over ATL.

## Data Availability

The Irish dataset, accessed 8 February 2023, used in our work can be downloaded from https://archive-beta.ics.uci.edu/.

## Supplementary Materials

The following supporting information can be downloaded at: https://www.mdpi.com/article/10.3390/e25071090/s1. References [44,58,62] have been cited in the Supplementary Materials.

## Appendix A

To complete the numerical simulation task of the theoretical scheme, we used a variational quantum circuit with eight entangled qubits from [58] to implement $\hat{G}\left({\vec{\theta }}_{G}\right)$, $\hat{D}\left({\vec{\theta }}_{D}\right)$, and $\hat{T}\left({\vec{\theta }}_{T}\right)$. The quantum circuit is shown in Supplementary Materials Section S1. In our task, the register **Out G|R_t|s_** required two qubits. As the data sample was encoded by a pure state, **Bath G** did not need to generate entropy. The generator $\hat{G}\left({\vec{\theta }}_{G}\right)$, involving 15 variational parameters, could generate the data for this task. The register **Bath D** was not necessary. Therefore, $\hat{D}\left({\vec{\theta }}_{D}\right)$, involving 42 variational parameters, operated on three qubits; one qubit served as **Out D** and the other two belonged to **Out G|R_t|s_**. For the classifier $\hat{T}\left({\vec{\theta }}_{T}\right)$, we noted that **Bath T** was not necessary. Thus, $\hat{T}\left({\vec{\theta }}_{T}\right)$, which had 15 variational parameters, only operated on two qubits of **Out G|R_t|s_**. The registers **Label** and **Test** required only one qubit, respectively. An additional qubit was employed to store the information of the gradient. Therefore, the workspace of the whole scheme was composed of five qubits in total.

In this scenario, we trained the gradient steps for this scheme according to the updated rules of Equation (17). At the beginning of the training sequence, the parameters were randomly chosen. Similarly, we adopted RMSProp [68] to update the learning rate. The idea of RMSProp is to divide the learning rate of a weight by a running average of the magnitudes of recent gradients for that weight. With the help of RMSProp, the learning rate is largely updated in flat directions and finely tuned in steep directions. This speeds up the training process; it has shown a good adaptation of learning rates in different applications. Here, the initial learning rates of $\hat{G}$, $\hat{D}$, and $\hat{T}$ were set to 0.001, 0.001, and 0.005, respectively. The training progress terminated; after that, the cost function converged at an equilibrium point. The numerical simulation results are shown in Figure 2.

## References

[1] Bishop C.M. (2016). Pattern Recognition and Machine Learning.

[2] Jordan M.I., Mitchell T.M. (2015). Machine learning: Trends, perspectives, and prospects. Science.

[3] Yang Q., Zhang Y., Dai W., Pan S.J. (2020). Transfer Learning.

[4] Devlin J., Chang M.-W., Lee K., Toutanova K. (2019). BERT: Pre-training of Deep Bidirectional Transformers for Language Understanding. Proceedings of the NAACL-HLT.

[5] Tai L., Paolo G., Liu M. (2017). Virtual-to-real deep reinforcement learning: Continuous control of mobile robots for mapless navigation. Proceedings of the 2017 IEEE/RSJ International Conference on Intelligent Robots and Systems.

[6] Mahajan D., Girshick R., Ramanathan V., He K., Paluri M., Li Y., Bharambe A., Van der Maaten L. (2018). Exploring the limits of weakly supervised pretraining. Proceedings of the Computer Vision—ECCV 2018.

[7] Liu B., Wei Y., Zhang Y., Yan Z., Yang Q. Transferable contextual bandit for cross-domain recommendation. Proceedings of the AAAI Conference on Artificial Intelligence.

[8] Pan W., Xiang E., Liu N., Yang Q. Transfer learning in collaborative filtering for sparsity reduction. Proceedings of the AAAI Conference on Artificial Intelligence.

[9] Bousmalis K., Silberman N., Dohan D., Erhan D., Krishnan D. (2017). Unsupervised Pixel-Level Domain Adaptation with Generative Adversarial Networks. Proceedings of the 2017 IEEE Conference on Computer Vision and Pattern Recognition (CVPR).

[10] Bousmalis K., Irpan A., Wohlhart P., Bai Y., Kelcey M., Kalakrishnan M., Downs L., Ibarz J., Pastor P., Konolige K. (2018). Using Simulation and Domain Adaptation to Improve Efficiency of Deep Robotic Grasping. Proceedings of the 2018 IEEE International Conference on Robotics and Automation (ICRA).

[11] Shrivastava A., Pfister T., Tuzel O., Susskind J., Wang W., Webb R. (2017). Learning from Simulated and Unsupervised Images through Adversarial Training. Proceedings of the 2017 IEEE Conference on Computer Vision and Pattern Recognition (CVPR).

[12] Isola P., Zhu J.-Y., Zhou T., Efros A.A. (2017). Image-to-Image Translation with Conditional Adversarial Networks. Proceedings of the 2017 IEEE Conference on Computer Vision and Pattern Recognition (CVPR).

[13] Ganin Y., Ustinova E., Ajakan H., Germain P., Larochelle H., Laviolette F., Marchand M., Lempitsky V. (2017). Domain-Adversarial Training of Neural Networks.

[14] Kim T., Cha M., Kim H., Lee J.K., Kim J. (2017). Learning to discover cross-domain relations with generative adversarial networks. Proceedings of the 34th International Conference on Machine Learning.

[15] Yi Z., Zhang H., Tan P., Gong M. (2017). Dualgan: Unsupervised dual learning for image-to-image translation. Proceedings of the 2017 IEEE International Conference on Computer Vision (ICCV).

[16] Bousmalis K., Trigeorgis G., Silberman N., Krishnan D., Erhan D. (2016). Domain separation networks. Proceedings of the 30th Annual Conference on Neural Information Processing Systems.

[17] Tzeng E., Hoffman J., Saenko K., Darrell T. (2017). Adversarial Discriminative Domain Adaptation. Proceedings of the 2017 IEEE Conference on Computer Vision and Pattern Recognition (CVPR).

[18] Long M., Zhu H., Wang J., Jordan M.I. (2017). Deep transfer learning with joint adaptation networks. Proceedings of the 34th International Conference on Machine Learning.

[19] Pei Z., Cao Z., Long M., Wang J. (2018). Multi-Adversarial Domain Adaptation. Proceedings of the AAAI Conference on Artificial Intelligence.

[20] Goodfellow I., Pouget-Abadie J., Mirza M., Xu B., Warde-Farley D., Ozair S., Courville A., Bengio Y. (2020). Generative Adversarial Networks. Commun. ACM.

[21] Creswell A., White T., Dumoulin V., Arulkumaran K., Sengupta B., Bharath A.A. (2018). Generative Adversarial Networks: An Overview. IEEE Signal Process. Mag..

[22] Wang K., Gou C., Duan Y., Lin Y., Zheng X., Wang F.-Y. (2017). Generative adversarial networks: Introduction and outlook. IEEE/CAA J. Autom. Sin..

[23] Holt C.A., Roth A.E. (2004). The Nash equilibrium: A perspective. Proc. Natl. Acad. Sci. USA.

[24] Gu M., Wiesner K., Rieper E., Vedral V. (2012). Quantum mechanics can reduce the complexity of classical models. Nat. Commun..

[25] Pudenz K.L., Lidar D.A. (2013). Quantum adiabatic machine learning. Quantum Inf. Process..

[26] Biamonte J., Wittek P., Pancotti N., Rebentrost P., Wiebe N., Lloyd S. (2017). Quantum machine learning. Nature.

[27] Cai X.-D., Wu D., Su Z.-E., Chen M.-C., Wang X.-L., Li L., Liu N.-L., Lu C.-Y., Pan J.-W. (2015). Entanglement-Based Machine Learning on a Quantum Computer. Phys. Rev. Lett..

[28] Hentschel A., Sanders B.C. (2010). Machine Learning for Precise Quantum Measurement. Phys. Rev. Lett..

[29] Rebentrost P., Mohseni M., Lloyd S. (2014). Quantum Support Vector Machine for Big Data Classification. Phys. Rev. Lett..

[30] Li Z., Liu X., Xu N., Du J. (2015). Experimental Realization of a Quantum Support Vector Machine. Phys. Rev. Lett..

[31] Wossnig L., Zhao Z., Prakash A. (2018). Quantum Linear System Algorithm for Dense Matrices. Phys. Rev. Lett..

[32] Levine Y., Sharir O., Cohen N., Shashua A. (2019). Quantum Entanglement in Deep Learning Architectures. Phys. Rev. Lett..

[33] Dunjko V., Taylor J.M., Briegel H.J. (2016). Quantum-Enhanced Machine Learning. Phys. Rev. Lett..

[34] Temme K., Bravyi S., Gambetta J.M. (2017). Error Mitigation for Short-Depth Quantum Circuits. Phys. Rev. Lett..

[35] Cong I., Choi S., Lukin M.D. (2019). Quantum convolutional neural networks. Nat. Phys..

[36] Nguyen N., Chen K.-C. (2022). Bayesian Quantum Neural Networks. IEEE Access.

[37] Zhao Z., Pozas-Kerstjens A., Rebentrost P., Wittek P. (2019). Bayesian deep learning on a quantum computer. Quantum Mach. Intell..

[38] Li Y., Zhou R.-G., Xu R., Luo J., Hu W. (2020). A quantum deep convolutional neural network for image recognition. Quantum Sci. Technol..

[39] Amin M.H., Andriyash E., Rolfe J., Kulchytskyy B., Melko R. (2018). Quantum Boltzmann Machine. Phys. Rev. X.

[40] Kieferová M., Wiebe N. (2017). Tomography and generative training with quantum Boltzmann machines. Phys. Rev. A.

[41] Niu M.Y., Zlokapa A., Broughton M., Boixo S., Mohseni M., Smelyanskyi V., Neven H. (2022). Entangling Quantum Generative Adversarial Networks. Phys. Rev. Lett..

[42] Lloyd S., Weedbrook C. (2018). Quantum Generative Adversarial Learning. Phys. Rev. Lett..

[43] Hu L., Wu S.H., Cai W., Ma Y., Mu X., Xu Y., Wang H., Song Y., Deng D., Zou C. (2019). Quantum generative adversarial learning in a superconducting quantum circuit. Sci. Adv..

[44] Dallaire-Demers P.L., Killoran N. (2018). Quantum generative adversarial networks. Phys. Rev. A.

[45] Li T., Zhang S., Xia J. (2020). Quantum Generative Adversarial Network: A Survey. Mater. Contin..

[46] Kandala A., Mezzacapo A., Temme K., Takita M., Brink M., Chow J.M., Gambetta J.M. (2017). Hardware-efficient variational quantum eigensolver for small molecules and quantum magnets. Nature.

[47] Qi J., Tejedor J. (2022). Classical-to-quantum transfer learning for spoken command recognition based on quantum neural networks. Proceedings of the 2022 IEEE International Conference on Acoustics, Speech and Signal Processing (ICASSP).

[48] Mari A., Bromley T.R., Izaac J., Schuld M., Killoran N. (2020). ransfer learning in hybrid classical-quantum neural networks. Quantum.

[49] Gokhale A., Pande M.B., Pramod D. (2020). Implementation of a quantum transfer learning approach to image splicing detection. Int. J. Quantum Inf..

[50] Zen R., My L., Tan R., Hébert F., Gattobigio M., Miniatura C., Poletti D., Bressan S. (2020). Transfer learning for scalability of neural-network quantum states. Phys. Rev. E.

[51] Wang L., Sun Y., Zhang X. (2021). Quantum deep transfer learning. New J. Phys..

[52] Liu J., Zhong C., Otten M., Chandra A., Cortes C.L., Ti C., Gray S.K., Han X. (2023). Quantum Kerr learning. Mach. Learn. Sci. Technol..

[53] Haug T., Self C.N., Kim M.S. (2023). Quantum machine learning of large datasets using randomized measurements. Mach. Learn. Sci. Technol..

[54] Sheng Y.-B., Zhou L. (2017). Distributed secure quantum machine learning. Sci. Bull..

[55] Li W., Deng D.L. (2022). Recent advances for quantum classifiers. Sci. China Phys. Mech. Astron..

[56] Li W., Lu S., Deng D.L. (2021). Quantum federated learning through blind quantum computing. Sci. China Phys. Mech. Astron..

[57] Hu F., Wang B.-N., Wang N., Wang C. (2019). Quantum machine learning with d-wave quantum computer. Quantum Eng..

[58] Schuld M., Bocharov A., Svore K.M., Wiebe N. (2020). Circuit-centric quantum classifiers. Phys. Rev. A.

[59] Schuld M., Killoran N. (2019). Quantum Machine Learning in Feature Hilbert Spaces. Phys. Rev. Lett..

[60] Havlíček V., Córcoles A.D., Temme K., Harrow A.W., Kandala A., Chow J.M., Gambetta J.M. (2019). Supervised learning with quantum-enhanced feature spaces. Nature.

[61] Park G., Huh J., Park D.K. (2023). Variational quantum one-class classifier. Mach. Learn. Sci. Technol..

[62] Schuld M., Petruccione F. (2018). Supervised Learning with Quantum Computers.

[63] Peruzzo A., McClean J., Shadbolt P., Yung M.H., Zhou X.Q., Love P.J., Aspuru-Guzik A., O’Brien J.L. (2014). A variational eigenvalue solver on a photonic quantum processor. Nat. Commun..

[64] Liu H.L., Wu Y.S., Wan L.C., Pan S.J., Qin S.J., Gao F., Wen Q.Y. (2021). Variational quantum algorithm for the Poisson equation. Phys. Rev. A.

[65] McClean J.R., Romero J., Babbush R., Aspuru-Guzik A. (2016). The theory of variational hybrid quantum-classical algorithms. New J. Phys..

[66] Blank C., Da Silva A.J., De Albuquerque L.P., Petruccione F., Park D.K. (2022). Compact quantum kernel-based binary classifier. Quantum Sci. Technol..

[67] Yang Z., Zhang X.D. (2020). Entanglement-based quantum deep learning. New J. Phys..

[68] Xu D., Zhang S., Zhang H., Mandic D.P. (2021). Convergence of the RMSProp deep learning method with penalty for nonconvex optimization. Neural Netw..

