# Supplementary material for: Quantum Adversarial Transfer Learning
Source: Entropy (Basel). 2023 Jul 20;25(7):1090. doi: 10.3390/e25071090 (PMC10378263; doi:10.3390/e25071090)
Supplement: Supplementary file 1 [file entropy-25-01090-s001.zip › entropy-2413046-supplementary.pdf]

Supporting Information for Quantum adversarial transfer learning

Longhan Wang[1], Yifan Sun[1] and Xiangdong Zhang[1]

[1] Key Laboratory of advanced optoelectronic quantum architecture and measurements of Ministry of Education, Beijing Key Laboratory of Nanophotonics & Ultrafine Optoelectronic Systems, School of Physics, Beijing Institute of Technology, 100081, Beijing, China

E-mail: zhangxd@bit.edu.cn; yfsun@bit.edu.cn

SI: Scheme of quantum gradient

In order to obtain the gradient of the quantum circuit for the quantum cost function, we introduce the following quantum representation process. The calculation of quantum gradient has been applied in quantum generative adversarial networks (Ref. [44] in the main text) and quantum classifiers (Ref. [58] in the main text). Here, we modify the relevant theories and apply them in our scheme. The unitary transformation U parameterized is expressed as

$$U(\vec{\theta}) \equiv U_N(\vec{\theta}_N)U_{N-1}(\vec{\theta}_{N-1})...U_2(\vec{\theta}_2)U_1(\vec{\theta}_1) = \mu\prod_{j=1}^{N} U_j(\vec{\theta}_j)\,, \tag{S1}$$

where μ is the time-ordering operator, and the parameter vector $\vec{\theta}$ is the union of the parameter sub-vectors $\vec{\theta}_j$. It is convenient to introduce the ordered notation according to the definition of U

$$U_{a:b} \equiv U_a(\vec{\theta}_a)U_{a-1}(\vec{\theta}_{a-1})...U_{b+1}(\vec{\theta}_{b+1})U_b(\vec{\theta}_b) = \mu\prod_{j=b}^{a} U_j(\vec{\theta}_j)\,, \tag{S2}$$

$$U^{\dagger}_{b:a} \equiv U^{\dagger}_b(\vec{\theta}_b)U^{\dagger}_{b+1}(\vec{\theta}_{b+1})...U^{\dagger}_{a-1}(\vec{\theta}_{a-1})U^{\dagger}_a(\vec{\theta}_a) = \overline{\mu}\prod_{j=b}^{a} U^{\dagger}_j(\vec{\theta}_j)\,, \tag{S3}$$

where $\overline{\mu}$ is the anti-time ordering operator. Logically, we can denote $U(\vec{\theta}) = U_{N:1}$ and $U^{\dagger}(\vec{\theta}) = U^{\dagger}_{1:N}$.

We set that each $U_j(\vec{\theta}_j)$ can be represented by a single qubit gate or a two-qubit gate,

and that the parameter vectors of the quantum gate can be represented by $\vec{\theta}_j = (\alpha, \beta, \gamma, \kappa, \ldots)$. We define an initial state ρ_0, and the expectation value of the observable A estimated by $\vec{\theta}$ is given by

$$\langle A(\vec{\theta}) \rangle = tr[\rho_0 U^{\dagger}(\vec{\theta}) A U(\vec{\theta})]. \tag{S4}$$

Further, the gradient of Eq. (S4) relative to $\vec{\theta}$ can be written as

$$\frac{\partial}{\partial \vec{\theta}_j} \langle A(\vec{\theta}) \rangle = tr(\rho_0 (\partial_{\vec{\theta}_j} U^{\dagger}(\vec{\theta})) A U(\vec{\theta}) + \rho_0 U^{\dagger}(\vec{\theta}) A (\partial_{\vec{\theta}_j} U(\vec{\theta}))). \tag{S5}$$

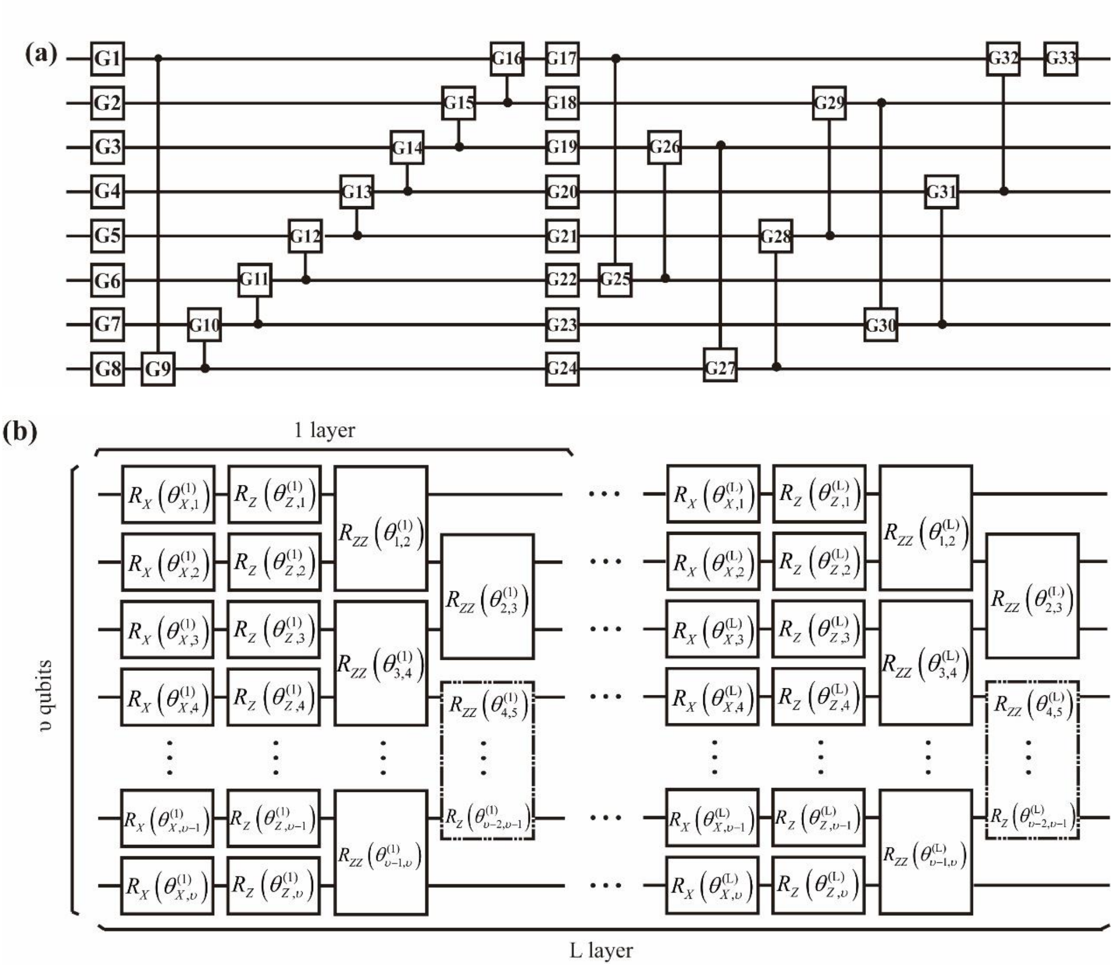

Figure S1. (a) Rapidly entangling eight-qubit circuit of depth 19, size 33. Horizontal wires correspond to qubits. All $Gj, j = 1, \ldots, 33$ are(parameterized) single-qubit quantum gates with trainable parameters. The $G1, \ldots, G8, G17, \ldots, G24$ and $G33$ are referred to as "local gates." The remaining gates form two cycles of *controlled* gates that are known to affect the entanglement entropy of the quantum states they act upon. **(b)** A practical circuit ansatz for the generator *G,* the discriminator *D* and the classifier *T* composed of *L* layers acting on *v* qubits. Each layer is composed of single-qubit *X* rotations parametrized

by angles $\vec{\theta}_X^l = (\vec{\theta}_{X,1}^l, ..., \vec{\theta}_{X,\upsilon}^l)$ followed by Z rotations parametrized by $\vec{\theta}_Z^l = (\vec{\theta}_{Z,1}^l, ..., \vec{\theta}_{Z,\upsilon}^l)$. A layer of two staggered sets of nearest-neighbor ZZ rotations parametrized by $\vec{\theta}_{ZZ}^l = (\vec{\theta}_{1,2}^l, ..., \vec{\theta}_{\upsilon-1,\upsilon}^l)$ follows the single-qubit rotations. The quantum circuit is universal for quantum computing since it can generate arbitrary single-qubit gates as well as entangling two-qubit gates.

In this paper, we apply the above conclusions to two special quantum gate circuits. In the section of Example, we use an entangling eight-qubit circuit which is composed of a single qubit gate and a two-qubit controlled gate in Fig. S1. (a) (Ref. [58] in the main text). The single qubit gate (both "local" and in controlled position) has the form

$$U(\alpha,\beta,\gamma) = \begin{pmatrix} e^{i\beta}\cos(\alpha) & e^{i\gamma}\sin(\alpha) \\ -e^{-i\gamma}\sin(\alpha) & e^{-i\beta}\cos(\alpha) \end{pmatrix}. \tag{S6}$$

Then Eq. (S5) can be transformed into the form

$$\frac{\partial}{\partial\vec{\theta}_j}\left\langle A(\vec{\theta})\right\rangle = \sum_{j=1}^{N} tr(\rho_0 U_{1:j-1}^\dagger(\partial_{\vec{\theta}_j} U^\dagger(\vec{\theta}_j))U_{j+1:N}^\dagger AU(\vec{\theta}) + \rho_0 U^\dagger(\vec{\theta})AU_{N:j+1}(\partial_{\vec{\theta}_j} U(\vec{\theta}_j))U_{j-1:1}), \tag{S7}$$

where $\partial_{\vec{\theta}_j} U(\vec{\theta}) = \sum_{j=1}^{N}(U_{N:j+1}(\partial_{\vec{\theta}_j} U(\vec{\theta}_j))U_{j-1:1})$, $\partial_\alpha U(\vec{\theta}_j) = U(\alpha + \pi/2, \beta, \gamma)$, $\partial_\beta U(\vec{\theta}_j)$ $= \cos(\alpha)U(0, \beta + \pi/2, 0)$, and $\partial_\gamma U(\vec{\theta}_j) = \sin(\alpha)U(\pi/2, 0, \gamma + \pi/2)$. In this case, we would run a quantum circuit for each term in Eq. (S6) separately and collect the sum of all terms using the classical method. We also note that, in addition to the one we are using here, there are other approaches (Ref. [62] in the main text) of calculating the gradient of a quantum circuit.

In the next section SII, we use a practical circuit ansatz in Fig. S1(b) (Ref. [44] of the main text). We set that every quantum gate is generated by a Hamiltonian $H_j = H_j^\dagger$, and that every quantum gate has the form $U_j(\theta_j) = e^{-\frac{i}{2}\theta_j H_j}$. Then Eq. (S5) can be transformed into the following form

$$\frac{\partial}{\partial\theta_j}\left\langle A(\vec{\theta})\right\rangle = -\frac{i}{2}tr(\rho_0 U_{1:j}^{\dagger}[U_{j+1:N}^{\dagger} A U_{N:j+1}, H_j]U_{j:1}), \quad \text{(S8)}$$

where [..., ...] is the commutator. We introduce the theory of quantum gradient circuit in Ref. [44] of the main text, and the corresponding gradient can be sampled from the expectation value of Pauli operator Z such that

$$\left\langle Z\right\rangle_{Grad} = \Pr(\left| x_{Grad}\right\rangle = \left|1\right\rangle) - \Pr(\left| x_{Grad}\right\rangle = \left|0\right\rangle) = \frac{\partial}{\partial\theta_j}\left\langle A(\vec{\theta})\right\rangle, \quad \text{(S9)}$$

in which the subscript *Grad* represents the register (denoted by register **Grad**) where the operator Z is sampled. When computing gradients, the operator A corresponds to the operator Z and the operator $\left|\lambda\right\rangle\left\langle\lambda\right|$. Next, we decomposed the generator $\hat{G}$, the discriminator $\hat{D}$, and the classifier $\hat{T}$ into N_G, N_D, and N_T gates respectively, such that

$$\begin{aligned}
&\hat{G}(\vec{\theta}_G) = G_{N_G:1}, \\
&\hat{D}(\vec{\theta}_D) = D_{N_D:1}, \\
&\hat{T}(\vec{\theta}_T) = T_{N_T:1}.
\end{aligned} \quad \text{(S10)}$$

We use Eq. (S8) and Eq. (S10) to represent the gradient of $\hat{D}$. This can be written as

$$\begin{aligned}
\frac{\partial}{\partial\theta_{Dj}}V(\vec{\theta}_G,\vec{\theta}_D,\vec{\theta}_T) = -\frac{i}{8M}\sum_{k=1}^{M} tr\Big((\rho_k^{R_t} - \rho_k^{G}(\vec{\theta}_G, z))U_{D,1:j}^{\dagger} \\
\times[U_{D,j+1:N_D}^{\dagger} Z U_{D,N_D:j+1}, H_j^D]U_{D,j:1}\Big).
\end{aligned} \quad \text{(S11)}$$

According to the theory of quantum gradient circuit in Ref. [44] of the main text, by attaching appropriate the NOT gate *X*, $X = \begin{bmatrix} 0 & 1 \\ 1 & 0 \end{bmatrix}$, the Hadamard gate *H* and the single-qubit *W* gate, $W = e^{-i\frac{\pi}{4}X}$. The quantum circuit of Eq. (S11) can be expressed in Fig. S2(a). Similarly, we can use the above gradient theory to obtain the gradient of the classifier. This is given by

$$\begin{aligned}
\frac{\partial}{\partial\theta_{Tj}}V(\vec{\theta}_G,\vec{\theta}_D,\vec{\theta}_T) = \frac{i}{2M}\sum_{k=1}^{M} tr\Big((\rho_k^{G}(\vec{\theta}_G, z) + \rho_k^{R_s})U_{T,1:j}^{\dagger} \\
\times[U_{T,j+1:N_T}^{\dagger}\left|\lambda_k\right\rangle\left\langle\lambda_k\right| U_{T,N_T:j+1}, H_j^T]U_{T,j:1}\Big),
\end{aligned} \quad \text{(S12)}$$

and the quantum circuit of Eq. (S12) is shown in Fig. S2(b). Further, the gradient of the generator can be expressed as

$$\frac{\partial}{\partial\theta_{Gj}}V(\vec{\theta}_G,\vec{\theta}_D,\vec{\theta}_T)=\frac{\partial}{\partial\theta_{Gj}}\Big[-\tfrac{1}{M}\sum_{k=1}^{M}\Big(-\tfrac{1}{4}tr(ZU_D(\vec{\theta}_D)U_G(\vec{\theta}_G)\rho_k{}^{R_s}U_G^{\dagger}(\vec{\theta}_G)U_D^{\dagger}(\vec{\theta}_D))$$
$$+tr(|\lambda_k\rangle\langle\lambda_k|U_T(\vec{\theta}_T)U_G(\vec{\theta}_G)\rho_k{}^{R_s}U_G^{\dagger}(\vec{\theta}_G)U_T^{\dagger}(\vec{\theta}_T))\Big)\Big].$$

(S13)

We set $V_1(\vec{\theta}_G,\vec{\theta}_D)=-\frac{1}{4M}\sum_{k=1}^{M}tr(ZU_D(\vec{\theta}_D)U_G(\vec{\theta}_G)\rho^{R_s}U_G^{\dagger}(\vec{\theta}_G)U_D^{\dagger}(\vec{\theta}_D))$ and $V_2(\vec{\theta}_G,\vec{\theta}_T)$ $=-\frac{1}{M}\sum_{k=1}^{M}tr(|\lambda_k\rangle\langle\lambda_k|U_T(\vec{\theta}_T)U_G(\vec{\theta}_G)\rho_k{}^{R_s}U_G^{\dagger}(\vec{\theta}_G)U_T^{\dagger}(\vec{\theta}_T))$, such that we respectively obtain the gradient of $V_1(\vec{\theta}_G,\vec{\theta}_D)$ and $V_2(\vec{\theta}_G,\vec{\theta}_T)$

$$\frac{\partial}{\partial\theta_{Gj}}V_1(\vec{\theta}_G,\vec{\theta}_D)=\frac{i}{8M}\sum_{k=1}^{M}tr\Big(\rho^{Rs}(z)U_{G,1:j}^{\dagger}[U_{G,j+1:N_G}^{\dagger}$$
$$\times U_D^{\dagger}(\vec{\theta}_D)ZU_D(\vec{\theta}_D)U_{G,N_G:j+1},H_j^G]U_{G,j:1}\Big),\qquad\text{(S14)}$$

$$\frac{\partial}{\partial\theta_{Gj}}V_2(\vec{\theta}_G,\vec{\theta}_T)=\frac{i}{2M}\sum_{k=1}^{M}tr\Big(\rho_k{}^{Rs}(z)U_{G,1:j}^{\dagger}[U_{G,j+1:N_G}^{\dagger}$$
$$\times U_T^{\dagger}(\vec{\theta}_T)|\lambda_k\rangle\langle\lambda_k|U_T(\vec{\theta}_T)U_{G,N_G:j+1},H_j^G]U_{G,j:1}\Big).\qquad\text{(S15)}$$

By attaching some appropriate quantum gates, we can obtain the quantum circuit shown in Fig. S2(c) and (d).

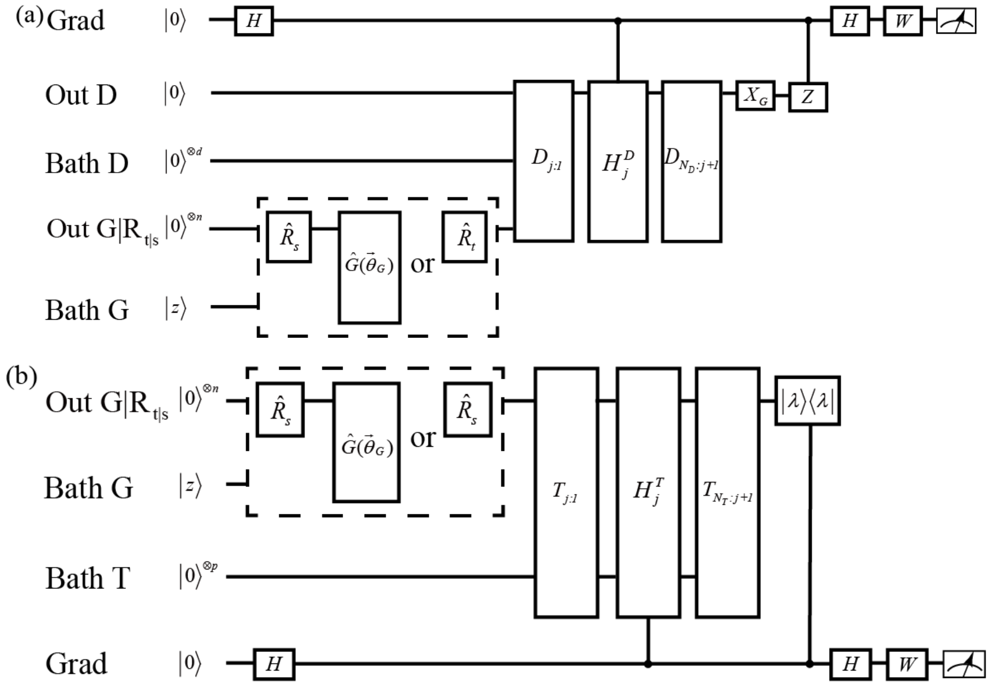

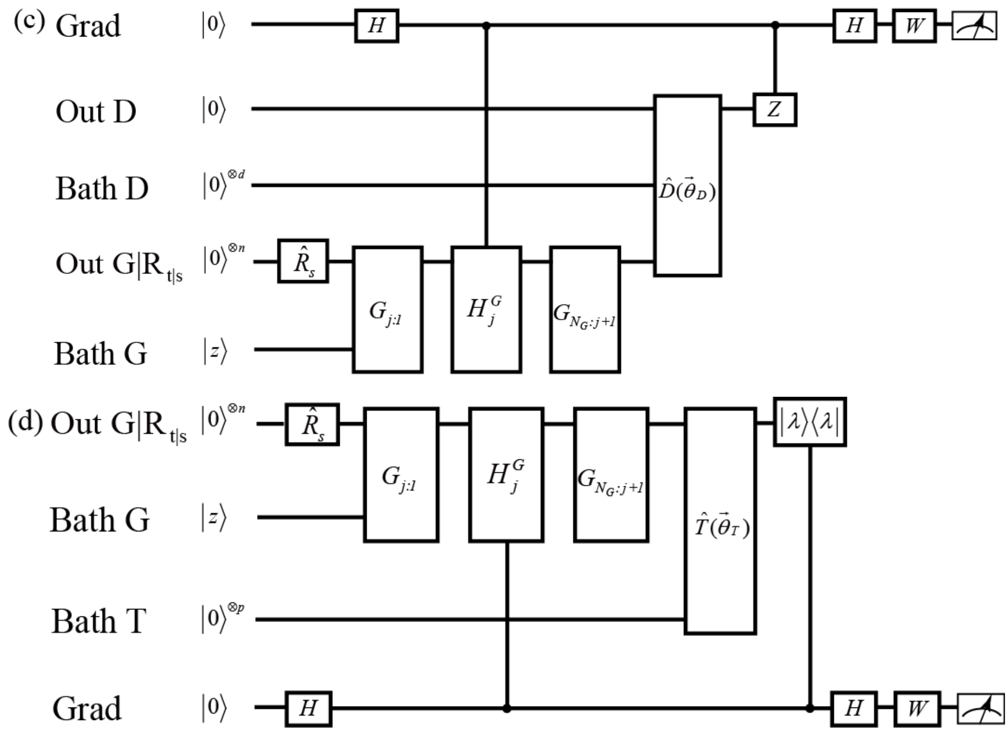

Figure S2. (a) Quantum circuit used to measure the gradient of the discriminator. (b) Quantum circuit for measuring the gradient of the classifier. (c) Quantum circuit used to measure the gradient of $V_1(\vec{\theta}_G, \vec{\theta}_D)$ in the generator. (d) Quantum circuit used to measure the gradient of $V_2(\vec{\theta}_G, \vec{\theta}_T)$ in the generator.

Next, we discuss the complexity of the quantum circuit of gradients. In the first case, we apply the properties of the scheme to Eq. (S7), $\partial_{\vec{\theta}_j} U(\vec{\theta})$ can be expressed by some linear combination of $O(N)$ unitary quantum circuits with the same structure. Then, we run a quantum circuit for each item in Eq. (S7), and its complexity is $O(N^2)$. In the second case, our quantum scheme adds a single qubit register **Grad** to the quantum circuit in Fig. S1(c). For the gradient of each parameter, we can use $O(N)$ unitary quantum circuits with the same architecture to realize it.

SII: Numerical simulation of a toy example

In this scenario, we perform a binary classification task of the states on a Bloch sphere, that is, to classify which quadrant the states on the Bloch sphere belong to. We select data samples on the equatorial plane of the Bloch sphere, that is, the states whose angle relative to

the z-axis is $\pi/2$. On the equatorial plane, starting from the positive direction of the x-axis, one data point is selected for each radian of the interval $\pi/100$, so that we can get a total of 200 data samples. We use the strategy of amplitude coding to represent 200 data samples in the form of density matrix. Then, we start from the positive x-axis and divide the coordinate system into the first, second, third, and fourth quadrants in the counterclockwise direction. We label the density matrices of the first quadrant with $|0\rangle\langle 0|$, and the density matrices of the third quadrant with $|1\rangle\langle 1|$. We call the set of density matrix and corresponding labels in the first and third quadrants the source domain dataset. The set of density matrices in the second and fourth quadrants is called the target domain dataset. Each dataset has 100 data samples. Based on the above datasets, we use the quantum circuit in Ref. [44] of the main text to implement $\hat{G}(\vec{\theta}_G)$, $\hat{D}(\vec{\theta}_D)$ and $\hat{T}(\vec{\theta}_T)$, respectively. We optimize the quantum cost function by adjusting the parameters $\vec{\theta}_G$, $\vec{\theta}_D$ and $\vec{\theta}_T$ in each circuit, and use this to verify our scheme.

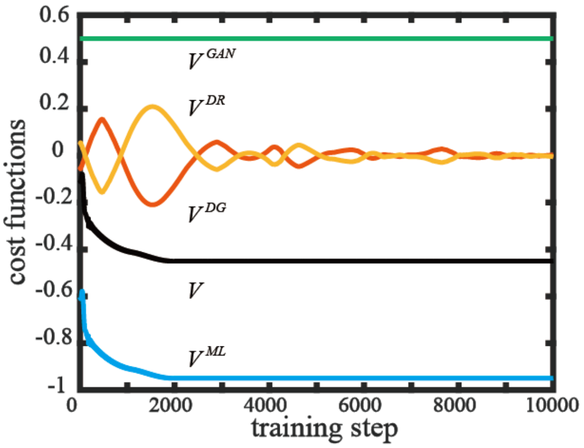

Figure S3 The values of quantum cost functions as a function of training step on Iris dataset. The V is the total cost function. The V^{ML}, V^{GAN}, V^{DG} and V^{DR} represent various components of the cost function.

In this task, the register **Out G|R$_{t|s}$** requires one qubit. Since the data sample is encoded by a pure state, the **Bath G** does not need to generate entropy. The generator $\hat{G}(\vec{\theta}_G)$ involving 4 variational parameters can generate the data for this task. The register **Bath D** is at least one qubit here. Therefore, $\hat{D}(\vec{\theta}_D)$, involving 32 variational parameters, operates on three qubits:

one qubit severs as the **Out D**, one qubit severs as the **Bath D**, the another one belongs to **Out G|R$_{t|s}$.** For the classifier $\hat{T}(\vec{\theta}_T)$, we find that **Bath T** need one qubit here. Thus, $\hat{T}(\vec{\theta}_T)$, which has 10 variational parameters, operates on two qubits: one qubit severs as the **Bath T**, the other one belongs to **Out G|R$_{t|s}$.** The register **Label** and **Test** here requires only one qubit respectively. An additional qubit is employed to store the information of gradient. Therefore, the workspace of the whole scheme is composed of seven qubits in total.

In this scenario, we train 10,000 gradient steps for this scheme according to the update rules of Eq. (S17). We also adopt RMSProp to update the learning rate. The initial learning rates of $\hat{G}$, $\hat{D}$ and $\hat{T}$ were set to 0.001, 0.0005 and 0.001, respectively. As shown in Fig. S3, we also plot the cost function V and its components, which are defined in the first example. We find that the discriminator $\hat{D}$ finally cannot determine whether the given data is fake data from the generator or data from the target domain, that is, V^{GAN} is stable at $1/2$. The classifier T is well trained, and the probability that the data obtains the correct label, V^{ML}, reaches -0.953. Finally, we select the data sample every interval $\pi/240$ in the second and fourth quadrants, and a total of 240 data samples are selected as the test data. Our classification accuracy reaches 100%. The results show that an equilibrium point exists for the cost function we propose. Meanwhile, the gap for knowledge transfer between the differently distributed datasets is effectively bridged, which shows that our scheme can effectively transfer knowledge with the generative model and adversarial training.
